# The influence of quality and respectful care on the uptake of skilled birth attendance in Tanzania

**DOI:** 10.1186/s12884-020-03278-z

**Published:** 2020-11-11

**Authors:** Myrrith Hulsbergen, Anke van der Kwaak

**Affiliations:** 1 Royal Tropical Institute (KIT), Amsterdam, the Netherlands; 2 Women’s Healthcare Center (WHC), Amsterdam, the Netherlands; 3 Vrije Universiteit (VU), Amsterdam, the Netherlands

**Keywords:** Quality of maternal care, (dis)respect, Tanzania, Skilled birth attendance, Bypassing, ICT, Interventions

## Abstract

**Background:**

An increase in the uptake of skilled birth attendance is expected to reduce maternal mortality in low- and middle-income countries. In Tanzania, the proportion of deliveries assisted by a skilled birth attendant is only 64% and the maternal mortality ratio is still 398/100.000 live births. This article explores different aspects of quality of care and respectful care in relation to maternal healthcare. It then examines the influence of these aspects of care on the uptake of skilled birth attendance in Tanzania in order to offer recommendations on how to increase the skilled birth attendance rate.

**Methods:**

This narrative review employed the “person-centered care framework for reproductive health equity” as outlined by Sudhinaraset (2017). Academic databases, search engines and websites were consulted, and snowball sampling was used. Full-text English articles from the last 10 years were included.

**Results:**

Uptake of skilled birth attendance was influenced by different aspects of technical quality of maternal care as well as person-centred care, and these factors were interrelated. For example, disrespectful care was linked to factors which made the working circumstances of healthcare providers more difficult such as resource shortages, low levels of integrated care, inadequate referral systems, and bad management. These issues disproportionately affected rural facilities. However, disrespectful care could sometimes be attributed to personal attitudes and discrimination on the part of healthcare providers. Dissatisfied patients responded with either quiet acceptance of the circumstances, by delivering at home with a traditional birth attendant, or bypassing to other facilities. Best practices to increase respectful care show that multi-component interventions are needed on birth preparedness, attitude and infrastructure improvement, and birth companionship, with strong management and accountability at all levels.

**Conclusions:**

To further increase the uptake of skilled birth attendance, respectful care needs to be addressed within strategic plans. Multi-component interventions are required, with multi-stakeholder involvement. Participation of traditional birth attendants in counselling and referral can be considered. Future advances in information and communication technology might support improved quality of care.

## Background

Increasing uptake of skilled birth attendance is expected to reduce maternal mortality by preventing complications during childbirth, or enabling early treatment of any such complications [1, 2]. Additionally, it is believed that low quality of care is the main cause of low uptake of skilled birth attendance by childbearing women [3]. The World Health Organization (WHO) defines quality of care as “the extent to which healthcare services provided to individuals and patient populations improve desired health outcomes” [3].

Globally, uptake of skilled birth attendance was around 81% in 2017, as compared to 68% in low- and middle-income countries [3, 4]. Trends in the prevalence of skilled birth attendance are upwards but unequal; in 2000 63% of births at the global level were attended by skilled staff compared to just 41% in sub-Saharan Africa [5]. Worldwide the maternal mortality ratio (MMR) dropped from 342/100.000 live births in 2000 to 211/100.000 live births in 2017 [6]. The MDG 5 target aimed to reduce the MMR by 75%, and SDG target 3.1 aims at a reduction to less than 70/100.000 live births [7, 8]. To reach these global goals, an accelerated increase in skilled birth attendance needs to take place.

The United Republic of Tanzania is the largest country in the Eastern part of Africa, just south of the Equator. The population was estimated at 60 million in 2019, 70% of whom live in rural areas [9]. Literacy is low, with 23% of women unable to read or write and significant differences between the literacy levels of women living in urban (11%) and rural (30%) areas [10]. In 2015, 22.8% of the population lived below the poverty line of 1.90 USD/day [11]. The maternal mortality ratio (MMR) in Tanzania has reduced from 842/100.000 live births in 2000 to 398/100.000 live births in 2015. However, the MDG 5 target of a 75% reduction in MMR has not been met [12–14]. The most common causes of maternal death are complications during or after childbirth such as haemorrhage, hypertensive disorders, sepsis, complications due to abortions, thromboembolism, and obstructed labour. Of these complications, 70% are preventable [2, 15].

**Fig. 1:**
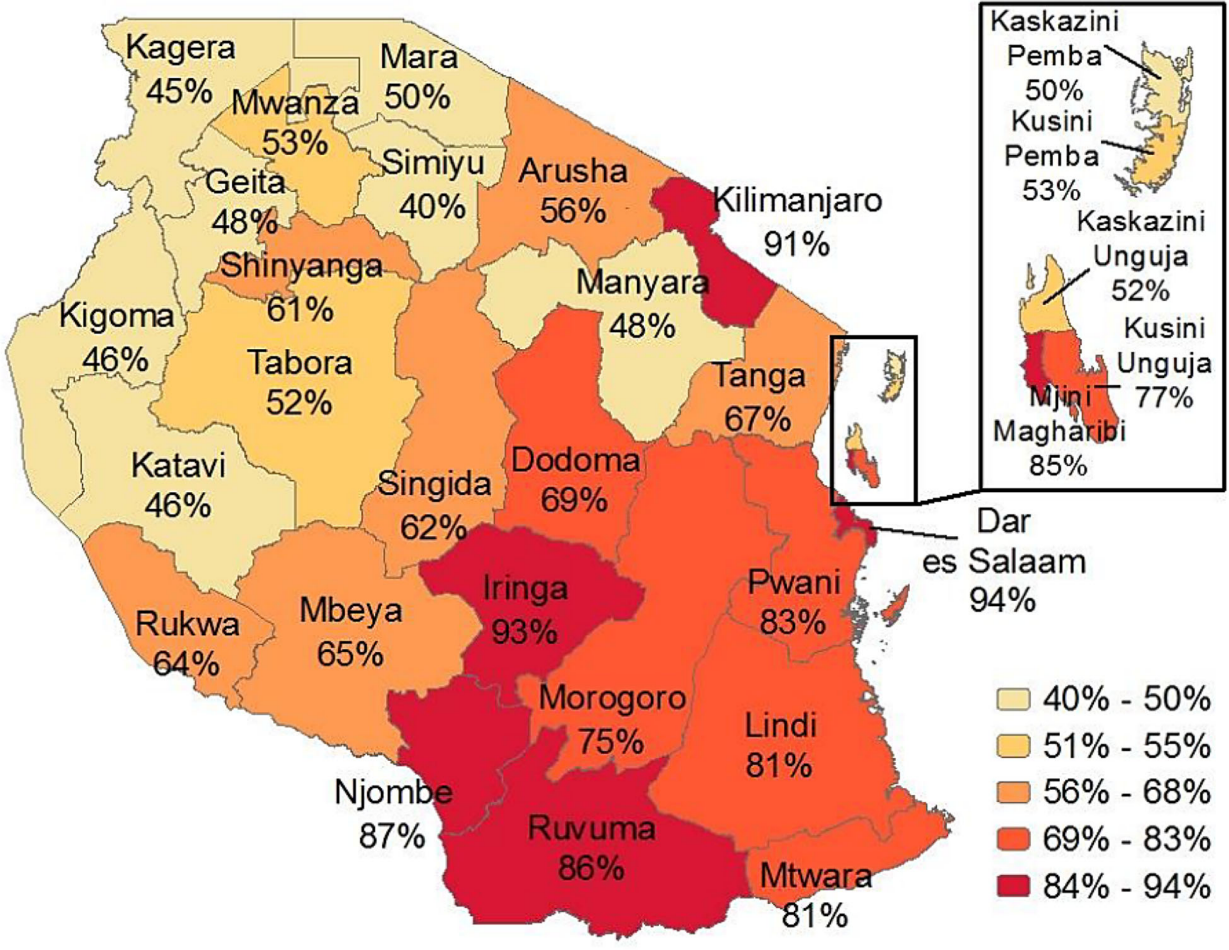
Skilled assistance at delivery by region *Percentage of live births in the 5 years before the survey assisted by a skilled provider; source DHS Tanzania (2015)*, www.dhsprogram.com [10]

In 2016 64% of deliveries were assisted by a skilled attendant, a significant increase from 1999 when this was the case for just 36% of deliveries [10]. However, the differences between the capital Dar es Salaam and rural areas are enormous: 95% of births in the capital are assisted by a skilled birth attendant, versus 42% in rural areas (Fig. 1, www.dhsprogram.com) [10]. Women often choose to deliver at home with a traditional birth attendant (TBA) or a family member who observes progress and facilitates the delivery [16–18].

Antenatal care (ANC) is attended by more than 95% of pregnant women in Tanzania at least once, but only just over 50% of women attended the four or more ANC visits recommended by the Focused Antenatal Care (FANC) guidelines in 2014 [10, 19]. Since 2016, the WHO has increased the recommended minimum of ANC visits to eight, as the FANC guidelines were based on the expectation that most pregnancies were uncomplicated. These more recent WHO recommendations aim to ensure improvements in continuity of care and communication between service provider and patient, as well as reductions in adverse perinatal outcomes [20].

In 2014 the Tanzanian health sector included 308 hospitals, 692 health centres and 6942 dispensaries [21]. Public primary healthcare (PHC) facilities serve as gatekeepers within the formal healthcare system. Although these have been extended and upgraded to improve coverage since independence, disparities still exist between rural and urban areas [10, 22, 23]. The number of medical personnel required to conform to WHO standards is 23 doctors, midwifes and nurses for every 10.000 people in a population. In Tanzania this proportion was only 3 medical professionals per 10.000 in 2010 [24]. Tanzania has no legal structure that regulates the registration of public health facilities, so there are no official minimum national standards on quality of care [16]. A five star-rating system was established in 2015 to gather information about the problems around access to and referral between health facilities. Results were confronting: care provided in healthcare facilities fell far below acceptable minimum standards [16, 21].

It is believed that poor quality of care is the main cause of inadequate uptake of skilled birth attendance [3]. But despite the Ministry of Health’s strategic plans and interventions to increase health coverage by improving quality of care and access, skilled birth attendance is still too low and maternal mortality too high [21, 25]. The WHO also discusses the importance of respectful maternal care in improving the uptake of skilled birth attendance, with respectful maternal care defined as “care organised for and provided to all women in a manner that maintains their dignity, privacy and confidentiality, ensures freedom from harm and mistreatment, and enables informed choice and continuous support during labour and child birth” [26]. Although many studies show that access to respectful maternal care in Tanzania could be significantly improved, this is not part of national strategic planning [27–35].

The objective of this article is to review the determinants of quality of care and respectful care in Tanzania, in order to identify the gaps in service delivery that result in low uptake of skilled birth attendance. The aim is to provide a range of recommendations on how to increase the uptake of skilled birth attendance, with a possible positive effect on the reduction of maternal mortality.

## Methods

This narrative review presents part of the unpublished thesis of the first author, titled “Factors influencing the uptake of skilled birth attendance in Tanzania; a literature review”. This complete review was performed from February to August 2019, and presented an analysis of: societal and community factors influencing the uptake of skilled birth attendance; health-seeking behaviour leading to the decision to seek care; and the quality of maternal care (including respectful care) in Tanzania. The stages of the complete literature review were consistent with these objectives and followed the different levels within the guiding framework as outlined below. For each level, a list of keywords was used. These could be overlapping between the different levels. Articles were selected using in- and exclusion criteria.

This narrative review only addresses the lowest level of the framework, a review of “Facility Quality”, originally the third objective of the above-mentioned thesis. Keywords used were: abuse, acceptance, access, advise, Africa, antenatal, attitude, best practice(s), bypassing, communication, counselling, delivery, discrimination, (dis)respect(ful), distrust, education, ethnicity, expectation, family, guidelines, e-/mHealth, health seeking (behaviour), integrated (care), intervention, maternal (care), outcome, measurement, perspective (client/ user), prevalence, protocols, quality, RCT, rights, RMC, skilled birth attendance, SBA, staff, (systematic) review, Tanzania, TBA, technology, uptake. AND/OR strategy was used depending on the results found.

To identify important articles and reports while avoiding an overly onerous process, peer-reviewed publications and grey literature from the last 10 years were reviewed. Due to time constraints which meant use of translator services was not feasible, only English language content was included. Where interesting publications of an earlier publication date were found by chance, these were included as well. Where the full text could not be retrieved, or the article was not in English, these were excluded.

PubMed, Medline, Research Gate and the online library of the VU University in Amsterdam were consulted, and the search engines Google and Google Scholar were used to find peer-reviewed articles. Grey literature was sourced from various websites: from the Ministry of Health of Tanzania, from unilateral organizations such as the WHO and World Bank, and from academic organizations such as Cochrane, the International Federation of Gynaecology and Obstetrics (FIGO), and the Association of Gynaecologists and Obstetricians of Tanzania (AGOTA). Lastly snowball sampling was used, identifying crucial references during the reading of selected articles. This is a non-probability sampling technique whereby selected articles provide referrals to other articles as required for the study. Even when these publications were published prior to the last 10 years, these were included.

**Fig. 2:**
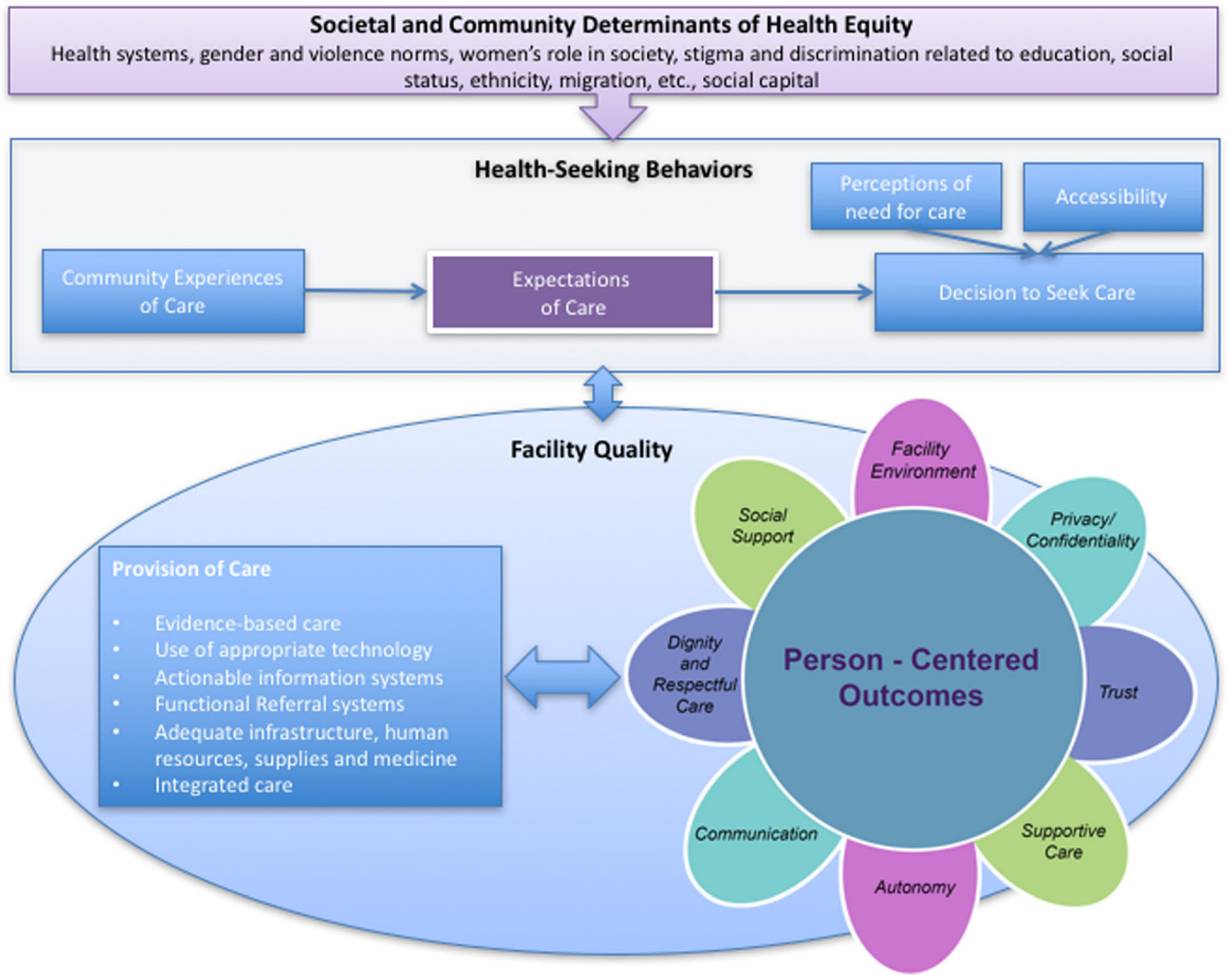
Person-centered care framework for reproductive health equity from Sudhinaraset (2017); *source* 10.12688/gatesopenres.12756.1, *original citation* [36]

Sudhinaraset’s person-centered care framework for reproductive health equity (Fig. 2, 10.12688/gatesopenres.12756.1) was used to evaluate the determinants of quality and respectful care in Tanzania in order to identify gaps in service delivery [36]. This framework was developed in 2017 to improve the quality of reproductive healthcare in low- and middle-income countries (LMIC) by emphasising the importance of patient-centeredness. It places a broad focus on health-seeking behaviour, and it pays particular attention to different patient-centred factors (including respectful care) in the lowest level of the framework.

## Results

As part of this narrative review, both qualitative and quantitative studies were considered. A range of qualitative studies was described, in addition to quantitative studies that were compared and described. Comparison of studies was complicated by differences in study type, selection criteria and study focus.

Results will be presented in relation to the question: which factors influence the uptake of skilled birth attendance within the context of Tanzania, and what lessons can be learned? Patients’ reactions to disrespectful and low-quality maternal care are described, as are best practices in respectful maternal care.

### Provision of care

A national cross-sectional study in 2015 determined the availability of Basic Emergency Obstetric and Neonatal Care services (BEmONC) in Tanzania [37]. Clinical guidelines were only available in less than 30% of facilities, and only approximately 20% of facilities had one or more trained providers. This low coverage, in combination with shortages of drugs and equipment, resulted in substandard quality of care and disparities between dispensaries, health centres and hospitals. Monitoring of quality, auditing of death cases, and evaluation of clients’ viewpoints were all significantly associated with better preparedness for BEmONC services [37].

### Availability of drugs and supplies

Since 1993, Medical Stores Department (MSD) has been delivering drugs and supplies to healthcare facilities in Tanzania. MSD works under the Ministry of Health semi-autonomously, and drugs and supplies need to be ordered via district medical officers. This supply chain is slow and unreliable in both rural and urban areas [27, 37, 38]. In addition, basic utilities like electricity and running water (provision of which is the government’s responsibility) are unreliable, especially in rural areas [10, 21]. Lack of drugs and equipment influences quality of care and can demotivate healthcare providers [27, 30, 32]. According to health workers in two studies, better availability of resources would encourage more pregnant women to deliver in health facilities rather than at home [27, 38].

### Human resources

Several studies discussed the lack of skilled healthcare providers in Tanzania [27, 38–40], which leads to untrained staff (15–18%) reportedly performing tasks at ANC visits for which they are not adequately qualified [38, 41]. Interestingly, in one retrospective study on integrating mother-to-child-transmission prevention services into existing services in five regions in Tanzania, staff workloads were investigated using a modification of the Workload Indicators of Staffing Need (WISN) method [41, 42]. Across 60 health facilities, the average workload was only around 50% of what was expected, but staffing levels were incomplete due to absenteeism and paid training leave [41]. This increased workloads for available, sometimes untrained health workers. Several studies have suggested that skilled birth attendants rarely attend in-service trainings, and that health workers feel lacking in knowledge and opportunities for career-progression [27, 37, 38, 43, 44].

Good management and role distribution were discussed in De Jongh’s review [45]. This review found that strong managers can improve accountability and retention of motivated workers, resulting in less corruption and improvements in equity within the health system [32, 45–47]. A good example of use of local accountability mechanisms was described in Tanga region, where healthcare workers carried out outreach to villages to address the health needs of pregnant women without financial support from district level government [27]. In contrast, an observational study in Dar es Salaam concluded that strong organization of the labour ward was lacking; midwives did not respond to clients’ needs and exhibited dishonest behaviour like writing false observations on checklists [32]. Leonard explored the difference in quality of maternal care between public facilities and those operated by NGOs in rural areas, which showed that NGO accountability mechanisms seem to play a positive role in good management [46].

### Evidence-based care

National clinical practice guidelines and initiatives such as “Focused Antenatal Care (FANC)”, “Emergency Obstetric Job Aide” and the “Antenatal Card” are inspired by WHO/UNICEF guidelines [3, 26, 43, 44, 48–51]. However, local availability and application of guidelines was often limited. The antenatal card is an exception to this, and is a widely used ANC guiding tool in several African countries including Tanzania [37, 43, 44, 51–54]. Several possible reasons were mentioned for the limited availability and use of guidelines, including the time-consuming nature of developing user-friendly guidelines, issues of distribution to all health facilities, and poor adherence of health workers to guidelines [51, 53].

### Referral system and actionable information systems

Neither basic nor comprehensive emergency obstetric and neonatal care is available in all settings, so a functioning patient referral system is essential [27, 37, 48]. This requires transport at low or no cost to reduce financial barriers to patient referral [2, 27].

Good communication between healthcare providers, TBAs, and the formal health system is also needed [17] since delay and reduced quality at recipient facilities is often the result of poorly communicated referrals [39]. The government supports information and communication technology (ICT), and many means of communication such as written letters, phone calls, SMS texting and telemedicine are now available in Tanzania [21, 39, 55, 56]. In Kigoma, Pwani and Morogoro regions telemedicine connects doctors from rural areas to medical specialists, which has been documented to increase uptake of teleconsultation, teleconferencing and e-learning [56, 57]. Community-based applications are also available: mHealth is used by community health workers in Singida Region to inform and counsel pregnant women, and SMS and internet messages provide information on health issues [58, 59]. A randomised controlled trial in Zanzibar explored the influence of SMS text messaging and a free-call system on ANC attendance and skilled birth attendance in PHC settings in Tanzania [60, 61]. This study recorded significant increases in skilled birth attendance for urban residents from 50–82% (AOR 5.73), and of repeated ANC visits from 31–44% (AOR 2.39). However, most of these communication strategies are only available in the context of interventional studies.

### Integrated care

Since more than 95% of women attend at least one ANC visit during their pregnancy, ANC offers opportunities for counselling, testing and information provision. This integration of care might increase uptake of repeated ANC visits and as a result the uptake of skilled birth attendance. “One-stop shop” facilities appear to be better attended by service users [21, 25, 38, 44, 45, 49, 62, 63]. The health system should ensure the presence of skilled attendants, equipment, financial resources and a good monitoring and evaluation system [21, 38, 45]. This is important as the effectiveness of integrated care can be significantly compromised by inadequate staffing levels or lack of supplies, as demonstrated by one study which looked at differences in provider knowledge and the delivery of messages to patients in Morogoro region [38].

### Person-centred care

The personal component of maternal care provision is seen to be highly important in determining how satisfied patients are with the quality of their care [27, 29–34, 36, 64–68]. Sudhinaraset highlights eight components that directly impact patient satisfaction, the components of which are generally described under the label: (dis)respect (Fig. 2). We also use this term as a superordinate denomination, and will focus on (dis)respect in the different elements of person-centred care in an in-depth way.

Disrespectful care can be seen as a form of power imbalance between patient and healthcare provider [29]. In several hospital-based studies in Dar Es Salaam and Tanga region in Tanzania, disrespect was measured in 15–70% of patients using direct observations and interviews [31–33, 68]. Repeated interviews some weeks after delivery found higher prevalence than at discharge, suggesting that after women had further reflected on their care after returning home, they appraised it more negatively [31, 66].

Lack of social support during labour was mentioned in several studies [27, 30, 34, 35, 40]. Healthcare providers in PHC settings in Tanga region admitted that there was no social support available and reported that they understood why women might prefer to deliver at home [27]. Arranging labour companionship was reportedly difficult due to the local healthcare culture which did not place importance on companionship. In addition, delivery rooms were often shared by several women in labour, which was not conducive to the presence of companions since levels of privacy were inadequate [27, 32]. Nevertheless, the availability of a birth companion appeared to be an important factor in improving healthcare worker attitudes, presumably as companions serve as potential witnesses to disrespectful care [40, 69, 70].

Despite the aforementioned barriers to provision of social support, some studies recorded that support was offered [30, 40]. In a missionary hospital in rural north-central Tanzania, social support was offered to and accepted by 23/25 women during the first stage of labour, with the advantage that the companion could inform nurses about the progression of labour [40]. Rosen’s international study reported that social support was offered to 22–43% of the 2164 observed women [30].

Issues relating to a lack of privacy in Tanzanian healthcare facilities were confirmed in several studies [27, 30–32]. The prevalence of lack of privacy reached 23–76% in the two largest observational studies, each including around 2000 women. Problems were reported mainly in relation to poor infrastructure of the labour room, in combination with loud voices and inappropriate language used by healthcare workers [30, 31].

Studies showed that some women were physically and psychologically abused. They were reportedly slapped or beaten and verbally intimidated or discriminated against [28, 30–32, 34, 71]. Difficulties meeting treatment costs resulted in delays to treatment or verbal discrimination [30, 34, 47]. Women often felt neglected due to verbal intimidation, or felt that their requests were ignored. Some women reported delivering their child without a skilled birth attendant present, or experiencing delivery complications that went unnoticed [30, 32, 34, 40, 47, 71]. Lack of informed consent was common before procedures, undermining the autonomy of women [30–32]. Harmful or dangerous procedures were even observed, such as suturing without analgesia, conscious administration of inadequate dosages of medication, and artificial rupture of membranes with a broken glass ampule [30–32].

During ANC visits, communication about risk factors, danger signs, delivery planning and skilled birth attendance seemed to be inadequate [19, 28, 30, 52, 63, 72]. This could be due to high staff workloads or lack of knowledge on the part of healthcare workers. Inadequate communication at ANC visits was described in a study in Ngorongoro district: instead of counselling all women towards skilled birth attendance, only women with risk factors were counselled. In addition, delivery planning was not discussed, and the importance of postnatal care was not clearly communicated [28]. Women also feared unnecessary examinations and procedures and compulsory delivery positions, which were not openly discussed at ANC visits. Health workers responded that they were too busy to counsel women extensively, and blamed women’s fear on lack of knowledge and low educational levels [28].

### Patients’ reactions to disrespectful and low-quality maternal care

Several studies have described a range of possible reactions to poor quality maternal care: quiet acceptance of abuse, bypassing the health facility, or deliberate delivering at home with or without a TBA [22, 23, 28, 31, 34, 47, 54, 71].

Accepting disrespect can be the result of stigma and fear, of sympathy for staff working in high-pressure situations, or of normalisation of abuse. Women might fear that raising complaints could influence the future treatment they receive, or risk closure of the health facility, resulting in reduced access to healthcare [31, 34]. Cultural normalisation of power imbalances and violence against women may also contribute to the acceptance of disrespect or abuse, due to a lack of knowledge of acceptable standards of care and human rights. This normalisation can result in risky deliveries due to a reduction or delay of uptake of skilled birth attendance [31, 34, 73, 74].

Another possible coping mechanism is bypassing to avoid the healthcare facility where the woman experienced unsatisfactory treatment. Bypassing occurs when women present for delivery at a facility other than their nearest health centre or dispensary [23]. Studies have shown that disappointment in received care at their closest health facility leads to bypassing of health facilities in between 44–75% of women [18, 22, 23, 31, 34, 39, 54, 71]. Factors that were significantly associated with bypassing were: being over 35 years of age; having a low number of children (0–1); a previous stay in a maternal waiting home or previous complications; as well as low trust and perceived quality of care in the nearest facility [22, 23]. Despite the costs of bypassing (travel, care, and opportunity costs), no association was found with wealth [22, 23]. The availability of emergency obstetric and neonatal care (EmONC) signal functions was significantly associated with bypassing [23]. Every extra signal function available in the nearest health facility reduced the likelihood of that facility being bypassed by almost 50% [23].

Lastly, in response to disrespectful or abusive care, women might decide to deliver at home with a TBA or family member, accepting the risks of not being close to skilled birth attendance. The Lancet Global Health Commission stated that more than 50% of patients in LMIC decide not to seek healthcare due to inadequate care quality [54].

### Applying best practice in respectful maternal care from Tanzania and other country contexts to the Tanzanian context

Three studies in comparable settings were selected as demonstrating examples of best practice; one randomised controlled trial (RCT) in Tanzania and two pre-post intervention studies in Kenya and Tanzania without comparison-groups [75–77]. All three studies implemented multiple interventions and ensured accountability of special quality-improvement teams through hospital staff and/or multi-stakeholder involvement. As the studies introduced multiple interventions simultaneously, combined outcomes are described [75–77].

### Interventions

Some documented interventions on the *improvement of birth preparedness* are: open days and workshops for the community, education about women’s rights, introduction of birth plans, improving access to information, group-wise pre-natal care, improving informed consent, and support in decision making [78, 79]. The RCT implemented a Client Service Charter (CSC); an existing, but un-implemented national charter on patient and provider rights and responsibilities [75, 80]. Multiple stakeholders further developed the CSC, and implementation was facilitated through meetings and workshops between the community and providers [75]. The two pre-post intervention studies implemented open birth days for pregnant women, and community workshops to improve access to information and communication with healthcare providers [76, 77].

Interventions on *attitude-improvement of healthcare workers* were attitudinal training, monitoring, and mentorship [78, 79]. The client-service chart, which was used as a birth preparedness tool in the RCT, was also used to increase healthcare providers’ knowledge of respectful maternal care, and to improve communication with clients [75]. The pre-post studies introduced respectful maternal care trainings, including mentorship and communication between providers on respectful care, as well as periodic observations of healthcare workers [76, 77].

The RCT focused on privacy in admission/discharge and delivery rooms, and on the weekly distribution of a list of shortages in the pharmacy as *infrastructure improvement* [75]. Small adjustments were also made, such as supplying tea for providers on duty. Respectful maternal care training resulted in the production of action plans, which addressed improvements to levels of privacy, changes to staffing structures, and improved payment of additional working hours [77].

### Outcomes of combined interventions

Outcomes were measured by interviewing women, and the pre-post studies added direct observations [75–77]. A reduction of disrespect and abuse was shown in all studies. The RCT showed a 66% reduction in the likelihood of experience of disrespect and abuse (CI 0.21–0.58). The two pre-post intervention studies also showed large reductions. Both respectful care (AOR 3.44, CI 2.45–4.84), and overall quality of care (AOR 6.19, CI 4.29–8.94) were graded as excellent in the RCT [75], and during observations and interviews improvements were noted in relation to privacy, physical abuse, detention, verbal abuse and confidentiality [76]. Patients and providers were very satisfied with the open birth days, and staff were positive about the RMC workshop and development of action plans to address barriers [77]. Knowledge of rights appeared to improve among both women and healthcare workers [77]. The interventions were supervised by a quality-improvement team comprised of healthcare workers, which increased accountability and trust [75, 76].

## Discussion

This study has evaluated the quality of maternal care and respectful care in Tanzania, in addition to outlining best practices in respectful care. This analysis was carried out in order to offer recommendations to the Ministry of Health in Tanzania and other stakeholders to increase the uptake of skilled birth attendance.

Ensuring both the availability of drugs and supplies and the proper auditing of deaths is vital to improving maternity care. Other important strategies include the improvement of: quality of in-service training, paths for career progression, referral systems, irregularities in human resources, and ICT infrastructure.

Shortages of drugs and supplies can be a higher-level problem due to inadequate supply chains, or due to a lack of management and training opportunities which renders staff knowledge of supply chains inadequate. Healthcare workers suffer from inadequate training and career-progression opportunities, in addition to shortages of resources and staff. However, one study suggested that, rather than lack of staffing per se, absenteeism and paid leave for training were the main factors driving low staffing levels. Absenteeism could be the result of low staff morale due to poor salaries, lack of training, and social circumstances in remote areas, in combination with management problems. Professional motivation can also be influenced by misalignment of work with skills. Ensuring full use of workers’ capabilities and integration of services can therefore improve knowledge and motivation [45, 46, 81].

In addition, increasing the use of ICT infrastructure within maternal care services may increase the motivation of healthcare providers. A strong health management and information system (HMIS) and the use of ICT for consultation with, or referral to other health facilities can improve quality of care. MHealth can be used for health education purposes, raising awareness, and reminders of ANC appointments. No interventional studies were yet identified on the influence of mHealth on women’s empowerment through messaging about women’s rights. In addition, communication infrastructure in Tanzania still needs to be extended to ensure that the no parts of the population are excluded.

Expectations about quality of maternal care in health facilities and about differences in supportive care between TBAs and healthcare workers influence the uptake of skilled birth attendance. TBAs may be able to play a role in connecting patients with health facilities, but most studies are from other low- and middle-income countries rather than Tanzania itself [82]. TBAs could refer women with risks during pregnancy and could serve as birth companions during labour in health facilities. However, this would require acceptance at multiple levels within the maternal healthcare system. It would need to be implemented into the PHC system, with training of TBAs and improvements in communication between TBAs and the formal healthcare system.

Disrespectful care is found in both rural and urban areas. It was described as a reaction to inadequate infrastructure, and shortages of staffing, drugs and supplies. However, shortages should never be an excuse to abuse and mistreat women. Cultural acceptance of inequality might increase the disrespectful behaviour and physical and psychological abuse experienced by women during delivery. No interventional studies were found on personal attitude measurement of (future) healthcare workers. Analysing the results of attitudinal tests might be more accurate than drawing conclusions from qualitative studies. Bradley’s systematic review evaluated the opinions of midwives on disrespectful care, but no studies were found which investigated the reasons behind disrespectful healthcare worker attitudes in Tanzania [29]. Futher research could inform decisions on the interventions needed to improve access to respectful care.

Bypassing the nearest facility or delivering at home as a response towards low quality and disrespect results is inefficient use of the existing healthcare system. In addition, bypassing can result in financial hardship for pregnant women and their families, so there is an urgent need to upgrade PHC facilities in terms of both availability and quality of EmONC services. Studies showed that these serve as pull factors and reduce bypassing. No studies included women who did deliver in their nearest health facility or at home, which is an opportunity for future research.

Multi-component interventions to improve respectful care demonstrate the best outcomes. Multi-stakeholder participation from national government to the community level is vital, to create ownership and sustainability by involving stakeholders in the development of programs, and in the implementation and analysis of research. At facility level, strong management, and facility-based quality-improvement teams are needed to ensure that interventions are implemented, results are monitored, and feedback is provided. Standards on privacy and social support to increase respectful care may require adjustment for local contexts. The success of implementation of forms of social support like birth companionship appears dependent on the acceptance of healthcare workers and women, as well as access to privacy in health facilities [70]. It is shown with high to moderate confidence that a companion helped pregnant women to understand information, gave practical and emotional support, and supported women in making their voices heard.

As availability and accessibility of facilities was lower in rural areas, more attention should be paid to these regions in the provision of care improvement. Disrespectful attitudes have been recorded in both rural and urban areas, so interventions on improvement of respect shown by healthcare workers towards women should be implemented in both rural and urban healthcare facilities at all levels within the healthcare system.

### Limitations of the framework

Sudhinaraset’s framework was used because of completeness of factors. However, some elements of this framework raised questions during the review. First, the WHO defines a health system as “all organizations, people and actions whose primary intent is to promote, restore or maintain health” (3). This implies that the health system directly influences health facility care quality through policies, provision of financial resources, and pre- and in-service trainings, which are missing aspects of the Sudhinaraset framework. Secondly, management and accountability are not described within the framework, but articles have shown that management is highly important. Management can both motivate health workers through clear task description and supervision, and implement interventions to improve RMC.

### Limitations of the study

One initial limitation was that the search for papers and articles focused on those written in English. This excluded literature written in the local language Kiswahili as well as other languages. Secondly, documents written more than 10 years ago were excluded, but some important documents may have been written before this. Through snowballing this study was able to include important literature from before 2009, however some useful documents might have been excluded.

Thirdly, studies showed strong heterogeneity on objectives, geographical location, size, variables of the study population, and study methods; hence conclusions should be drawn with caution. Fourthly, due to the focus on childbearing women in this study, ANC has not been explored more broadly. Lastly, bias due to normalisation of disrespectful care within the country context might have reduced the recorded prevalence of disrespectful care.

## Conclusion and recommendations

Conclusions are drawn from the results and discussion, and recommendations are offered to the Minister of Health & Social Welfare and the engaged local stakeholders on how to increase skilled birth attendance.

### Conclusions

Skilled birth attendance in Tanzania is still too low, and both the technical quality of care and poor provision of person-centred care seem to be major factors underlying this. However, respectful care is not mentioned in Tanzania’s strategic plans on improvement of equity and quality.

Shortages of supplies, human resources, referral systems, training, and opportunities for career progression all result in reduced quality and accessibility of maternal care. These negatively impact health workers’ motivation levels and increase disrespectful attitudes towards pregnant women. Abuse and discrimination towards patients might be the result of personal attitudes, or the acceptance of power inequalities within society, resulting in the normalisation of disrespectful behaviour towards pregnant women and vulnerable groups. Best practices in respectful care recommend multi-component interventions, multi-stakeholder participation and strong management to ensure implementation of interventions and sustainability of change. However, without the personal commitment of healthcare workers to changing attitudes, we are unlikely to see improvements in levels of respectful care provided to pregnant women. This suggests the value of the implementation of personal attitude measurements for (future) healthcare workers in upcoming research.

Lastly, PHC facilities need to be upgraded to ensure that the gatekeeper function is properly used. Collaboration with TBAs as identifiers of high-risk pregnancies and birth companions might increase uptake of skilled birth attendance in PHC. The application of new technologies, such as ICT in healthcare, can increase motivation of staff as well as improve access to healthcare.

### Recommendations

Recommendations are made at policy, implementation and research level. These recommendations require multi-stakeholder participation. Different interventions should be combined.

#### Recommendations on governmental policies and strategies

-The promotion of respectful care needs to be included in all national plans and programs to ensure accountability. To support respect for women in law, a Domestic Violence Act needs to be established and national policies need to be lined up with the Declaration of Human Rights.
-Public literacy needs to be increased and improvements in the education system need to be accelerated.

#### Recommendations on interventions

-Communication and two-directional understanding between communities and facilities needs to be improved by implementing “open birth days”. Community awareness of complications during pregnancy, respectful care and human rights can be increased through workshops and the involvement of voluntary health workers. This will empower women in decision-making and making their voices heard.
-The availability of good quality in-service trainings (on FANC, BEmONC/ CEmONC, RMC, birth companionship, attitudinal behaviour, and human rights) needs to be increased by expanding the amount of training sites and adding trainers.
-The curricula of pre-service education at medical, midwifery and nursing schools need to be adjusted by placing stronger emphasis on potential complications during pregnancy, attitudinal behaviour, and respectful maternal care.
-Job descriptions which highlight responsibilities for quality, respectful behaviour and birth companionship need to be guaranteed, and adherence needs to be ensured by mentorship, supervision and feedback-mechanisms to ensure professionalism, competency and commitment at all levels of healthcare work. This requires improvement of the infrastructure of antenatal clinics and labour wards (such as the availability of guidelines, drugs, equipment, and the possibility of ensuring patient privacy). Also, maternal and neonatal morbidity and mortality audits should be established, as an indication of improvements in quality of care.
-The antenatal clinic needs to be used as opportunity to inform women and their companions about birth preparation, danger signs, skilled birth attendance and birth companionship. Also, future contraceptive advice can be addressed. This requires sufficient staffing.
-Transportation and telecommunication systems should be improved, to form a sound referral system to higher levels of care.

#### Recommendations for further research

-Interventional research on personal attitude measurement of future healthcare workers as a mandatory test before pre-service training.
-Interventional research on personal attitude measurement of current healthcare workers.
-Interventional research on the role of TBAs as a bridge between pregnant women and the formal health care system (to improve counselling, birth preparation, referral, birth companionship, and postnatal care). This requires consensus between different stakeholders, and training of TBAs.

## Data Availability

not applicable.
